# Fabrication and Characterization of PDMS Waveguides for Flexible Optrodes

**DOI:** 10.1002/adhm.202304513

**Published:** 2024-04-28

**Authors:** Linda Rudmann, Daniel Scholz, Marie T. Alt, Alexander Dieter, Eva Fiedler, Tobias Moser, Thomas Stieglitz

**Affiliations:** ^1^ Laboratory for Biomedical Microtechnology Department of Microsystems Engineering University of Freiburg 79110 Freiburg Germany; ^2^ BrainLinks BrainTools University of Freiburg 79110 Freiburg Germany; ^3^ Institute for Auditory Neuroscience and InnerEarLab University Medical Center Göttingen 37075 Göttingen Germany; ^4^ Bernstein Center Freiburg University of Freiburg 79104 Freiburg Germany

**Keywords:** cochlea, in vivo, optogenetics, PDMS, waveguide

## Abstract

With the growth of optogenetic research, the demand for optical probes tailored to specific applications is ever rising. Specifically, for applications like the coiled cochlea of the inner ear, where planar, stiff, and nonconformable probes can hardly be used, transitioning from commonly used stiff glass fibers to flexible probes is required, especially for long‐term use. Following this demand, polydimethylsiloxane (PDMS) with its lower Young's modulus compared to glass fibers can serve as material of choice. Hence, the long‐term usability of PDMS as a waveguide material with respect to variations in transmission and refractive index over time is investigated. Different manufacturing methods for PDMS‐based flexible waveguides are established and compared with the aim to minimize optical losses and thus maximize optical output power. Finally, the waveguides with lowest optical losses (−4.8 dB cm^−1^ ± 1.3 dB cm^−1^ at 472 nm) are successfully inserted into the optogenetically modified cochlea of a Mongolian gerbil (*Meriones unguiculatus*), where optical stimuli delivered by the waveguides evoked robust neuronal responses in the auditory pathway.

## Graphical abstract

The demand for optical probes tailored to applications is ever rising for optogenetic research. Specifically, for applications such as the cochlea, transitioning from stiff glass fibers to flexible probes is required, especially for long‐term use. Polydimethylsiloxane with its low Young's modulus can serve as material of choice. Its long‐term usability as a waveguide material is investigated, and different manufacturing methods are established. The waveguides with lowest optical losses are successfully inserted into the optogenetically modified cochlea of a Mongolian gerbil (*Meriones unguiculatus*).

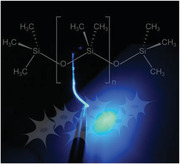

## 1 Introduction

**Figure 1:**
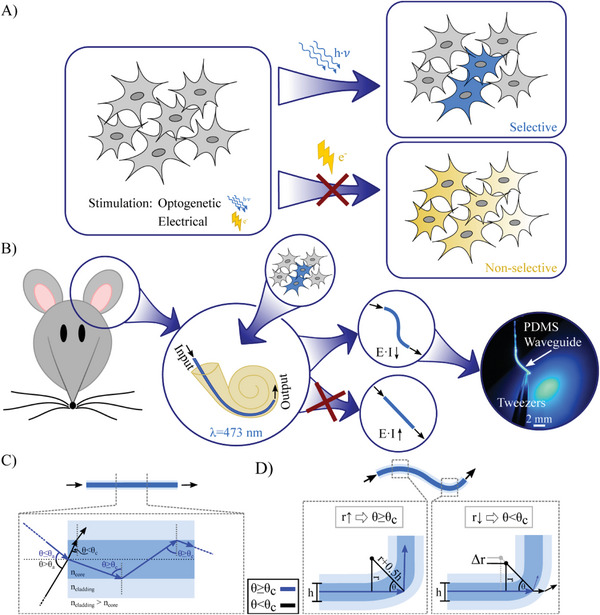
A) In contrast to electrical stimulation, optogenetics is capable of cell‐type specific neuronal control, which can be specified by promoter‐based expression. In addition, different opsins with distinct spectral properties can be combined. B) The presented silicone‐based flexible waveguides can be bent down to very small radii due to the low Young's modulus to access, e.g., the cochlea, while being connected to an external laser source. C) Schematic sketch of the working principle of a waveguide, with core refractive index *n*_core_ higher than the cladding refractive index *n*_cladding_. For angles *θ* at the core/cladding interface that are equal to or greater than the critical angle *θ*_c_, light is guided within the waveguide (blue ray). For external input angles greater than the acceptance angle *θ*_a_, total internal reflection (TIR) does not occur. D) Radiation loss in dependence of the bending radius *r*. For bending radii *r* exceeding the critical radius, the ray (blue) undergoes TIR at the interface (left). For smaller radii, radiation losses (black) occur (right).

The field of optogenetics emerged when bacteriorhodopsin, halorhodopsin and, in the early 2000s, channelrhodopsins 1 and 2 were identified and used for precise optical control of neuronal activity.^[^ ^1^ ^]^ Given its great impact on the life sciences, optogenetics was selected as the method of the year in 2010.^[^ ^2^ ^]^ Since then, it has become a standard tool in fundamental neuroscience to decipher brain function by interrogating molecularly defined neural circuits, something not possible with electrical stimulation (**Figure** **1**A). Whereas electrical stimulation unspecifically excites cells in the entire surrounding, optogenetically modified cells can be either excited or inhibited in a cell type‐specific manner depending on the opsin used.^[^ ^1^, ^3^ ^]^ In addition, users are not limited to the use of only one opsin, which further expands the possibilities with regard to research of complex neurological circuits.^[^ ^4^ ^]^ Worldwide, micro‐ and nanosystem engineers have developed various tools to deliver light by the means of light emitting diodes, laser diodes and waveguides to optogenetically modified target structures of the nervous system.^[^ ^5^, ^6^, ^7^ ^]^ Nowadays, optoelectronic neural probes have been mostly developed for acute and chronic fundamental neuroscientific research, and only recently approaches for translational research toward (human) clinical applications have been reported.^[^ ^6^, ^8^, ^9^ ^]^ Devices for clinical application have to meet medical device regulations eventually, e.g., FDA approval in the USA, TGA approval in Australia or Medical Device Regulation (MDR) for CE marking in countries of the European Union. Stability and longevity of the implant, transparency of light transmitting components and coatings, hermeticity of packages, and temperature stability (<2 K heating during operation) are target specifications that are often unmet in probes for preclinical studies.^[^ ^5^ ^]^

These specifications, as well as longevity and long‐term functionality (in terms of sufficient light delivery to the target site), need a more careful selection of materials, manufacturing and assembly approaches in translational devices as compared to tools for basic research, which is not a trivial task. Decoupling of the light source from light guiding structures is advantageous for long‐term applications with respect to safe operation of the optoelectronics and potential replacement of optoelectronic implantable pulse generators in which electronics and/or light sources need to be replaced independent from the implanted optoelectronic probe.

With respect to light guiding structures for optical implants, ordinary glass fibers are commonly used in optogenetic experiments.^[^ ^5^, ^10^, ^11^ ^]^ Yet, they are not designed to be operated in living tissue and hold a high Young's modulus *E* (*E*_Silica_ > 70 GPa),^[^ ^5^, ^12^ ^]^ especially when compared to the Young's moduli of the target sites, e.g., brain tissue (*E*_Tissue_ ≈ 1 kPa).^[^ ^5^ ^]^ Polymeric materials like poly(methyl methacrylate) (PMMA) or epoxy‐based photoresist SU‐8 can provide some degree of flexibility when dimensions are in the micrometer range;^[^ ^13^ ^]^ however, depending on the type of SU‐8 used those materials remain brittle in their stress–strain behavior^[^ ^14^ ^]^ without suitable support structures.^[^ ^15^, ^16^ ^]^ Silicone rubber or polydimethylsiloxane (PDMS) on the other hand is flexible due to its low Young's modulus *E*_PDMS_ of around 0.5 MPa,^[^ ^17^ ^]^ resulting in an over a factor 100 000 lower bending stiffness *E∙I* (moment of inertia *I*) than conventional glass fibers. In addition, it is one of the materials used in medical devices with a long history as substrate and packaging material that can be structured and integrated with different technologies.

Due to these properties and handling capabilities, PDMS was recently introduced as material for implantable waveguides (Figure 1B).^[^ ^18^, ^19^, ^20^ ^]^ Nevertheless, PDMS waveguides must prove that they transmit sufficient light to activate opsins in electrically excitable cells. Prerequisites are that they maintain their light transmission properties over time without getting opaque and no drastic refractive index changes occur, so that the core refractive index (*n*_core_) remains larger than the cladding refractive index (*n*_cladding_). These waveguides transmit light that enters at angles *θ* that are smaller than the acceptance angle *θ*_a_ due to total internal reflection (TIR), with the critical angle *θ*_c_ corresponding to the maximum *θ*_a_ (Figure 1C).^[^ ^21^ ^]^ Rays hitting a waveguide core being bend smaller than the critical radius *r*_c_ radiate out (Figure 1D).^[^ ^22^ ^]^ In addition, structural biocompatibility has to be proven in order for the probe to be considered for translational applications. In this study, we compare two different manufacturing technologies (laser‐structuring and molding) with respect to transmission properties and optical scattering. We then demonstrate that PDMS‐based waveguides can be used to elicit neuronal activity in the spiral ganglion of a genetically modified cochlea.

## 2 Results and Discussion

### 2.1 Waveguide Materials

Choosing an adequate material combination for the silicone‐based waveguides is crucial: First, it must be ensured that optical transmission of the relevant wavelength(s) is sufficiently high with the silicones that build core and cladding of the waveguides. Second, the materials that are used for core and cladding of the waveguides need a distinguishable refractive index difference in order to establish an optical interface where TIR occurs to ensure adequate light guiding. Third, besides the need for biocompatibility, investigations on the stability and longevity of the incorporated materials and their properties are inevitable with regards to translational research.

**Figure 2:**
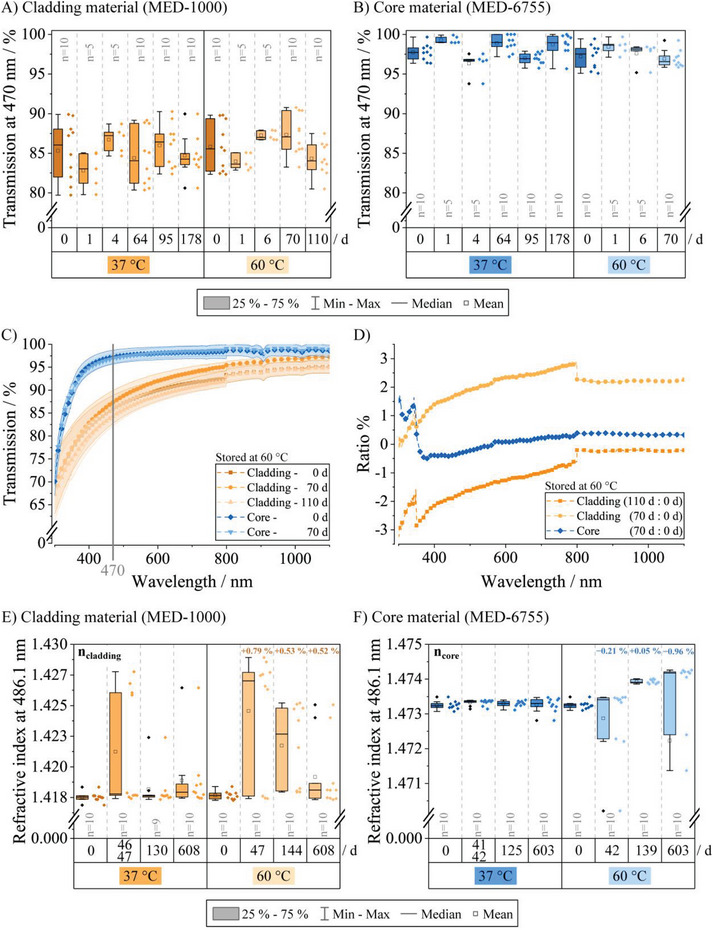
Transient transmission at 470 nm for the A) cladding and B) core material. Samples of both were stored at 37 °C for 178 d and up to 110 d at 60 °C. The data variation is in the low percentage range for both over the entire time and temperature span. The ranges of both *y*‐axes are chosen identically to better illustrate the different transmission properties. C) Wavelength‐dependent transmission (mean ± standard deviation) from 300 to 1100 nm of cladding (*n* = 10 each) and core (*n * =  10 each) samples stored at 60 °C for 110 and 70 d, respectively. The wavelength used for optogenetic activation of ChR2, 470 nm is depicted with a gray line. D) The ratio of aged materials with reference to pristine samples (cladding: 110 d/70 d:0 d; core: 70 d:0 d) confirms with its low percentage changes the stability of the materials across the entirety of the examined range. For clarity only every tenth data point is shown. Refractive indices of the E) cladding and F) core material at 486.1 nm (measurement temperature: 20 °C). All samples were stored either at 37 or at 60 °C for over 600 d. Maximum percentage refractive index changes with respect to the measured mean value at 0 d are indicated for 60 °C. Note the different *x*‐scaling in (E) and (F). One outlier (1.46) is not shown for the 60 °C condition at 603 d.

Here, we focused on the one‐component silicone Nusil MED‐1000 (cladding) and the two‐component silicone Nusil MED‐6755 (core), as these meet the requirements in terms of refractive index distinction and optical properties. In a first set of experiments, the stability of optical properties of these materials had been assessed by measuring the transmission (**Figure** **2**A–D) and refractive index (Figure 2E,F) over time.

Transmission through core and cladding material at 470 nm, a wavelength commonly used to activate the blue‐light gated ChR2, initially amounted to 85% ± 3% (mean ± standard deviation, *n* = 20, Figure 2A) for the cladding material, while the core material amounted to 97% ± 1% (mean ± standard deviation, *n* = 20, Figure 2B), respectively.

Samples were either stored at 37 °C, recreating body temperature, or at 60 °C to accelerate the overall ageing process.^[^ ^23^ ^]^ Despite some variability, the entire data ranged only ≈5% for the cladding (Figure 2A) and ≈3% for the core material (Figure 2B) when minimum and maximum values from the whole course of data acquisition were considered. The high stability of the chosen materials is further demonstrated by the comparison of optical transmission over the entire examined wavelength range from 300 to 1100 nm of samples stored at 60 °C for either up to 70 or 110 d, respectively (Figure 2C). This is further supported when the percent ratio is considered, where especially for the core material, changes only amounted to <1% for wavelengths >400 nm (Figure 2D). With this it can be stated, that both materials, cladding and core, retain their transparency over a time course of at least more than 500 d at 37 °C (110 d at 60 °C) and at least more than 300 d at 37 °C (70 d at 60 °C), respectively.

In addition to high light transmission, the refractive indices should not change to an amount where the TIR would be inflicted, and thus the waveguides would malfunction. Equally to transmission measurements, samples were stored at 37 and 60 °C after the initial measurements to evaluate their characteristics over an extended period of time. Refractive indices are presented at 486.1 nm and initially amounted to *n*_cladding_ = 1.417 and *n*_core_ = 1.473 (mean of *n* = 20 each) for cladding and core materials, respectively. The cladding material (Figure 2E) showed the highest data variability after 46 d/47 d at 37 °C, which then decreased again up to a storage time of 608 d in total. Data variability also decreased over time for the 60 °C samples after initially higher variations. For the core material, data variability at 60 °C is considerably higher in comparison to the 37 °C samples (Figure 2F). However, the data variation is not as drastic as it might appear. Values of the whole data set only vary around maximum 1% in total (*n* = 79/80 for cladding/core, respectively) (Figure 2E,F), allowing a refractive index independent functionality (*n*_core_ > *n*_cladding_) over an extended time period, since no values that would inflict their operation entirely were reached (cf. Figure 1C). Mean refractive index values amounted to 1.419 and 1.472 for *n*_cladding_ and *n*_core_ after 608 and 603 d at 60 °C, respectively. As long as the other requirements are met, functionality after more than eight years at physiological temperatures (37 °C) can be assumed from 60 °C data.

### 2.2 Fabrication

Choosing the right manufacturing approach is essential to fabricate reliable and high‐quality waveguides in a reproducible manner. The method of choice preferably maximizes optical output intensity, while being time‐ and cost‐efficient. We therefore investigated and compared two fundamentally different methods, both having their particular advantages and disadvantages which will be identified and evaluated. While the first method uses a laser‐processing approach, commonly used in rapid prototyping, the second method relies on microfabrication techniques to realize custom molds.

**Figure 3:**
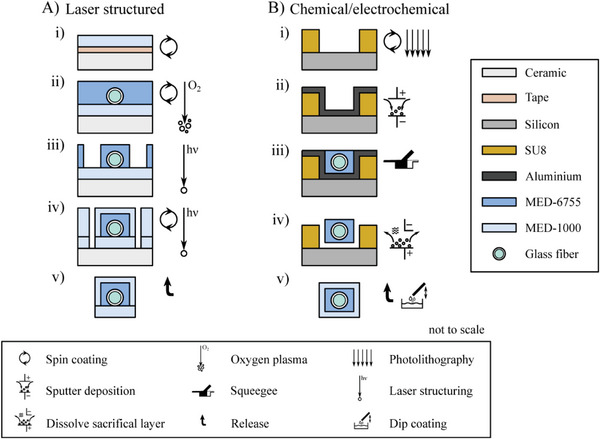
Development and comparison of the two different manufacturing processes for flexible silicone‐based waveguides. A) The simplified fabrication process based on laser structuring. i) The essential process starts with the spin‐coated bottom cladding silicone that was transferred to an alumina substrate. ii) Then, the core silicone is spin‐coated embedding glass fibers that are placed beforehand. iii) Next, the laser structuring of the core silicone, iv) followed by spin‐coating of the top cladding and subsequent laser structuring of the outline are performed. v) Finally, the finished waveguides are released. B) A schematic overview of the novel fabrication approach based on molding. i) The molds are fabricated with photolithography, ii) followed by a deposition of aluminum to serve as sacrificial layer. iii) Then the ferrules are placed into previously KOH etched V‐grooves (not shown) to align the glass fibers in the resist trenches and the core silicone is molded inside. iv) The sacrificial layer is etched, and v) the samples are released.

The rapid prototyping laser‐based approach is a subtractive manufacturing process, which starts with spin‐coating the cladding material onto a handling ceramic substrate (**Figure** **3**Ai). With the next step, glass fibers are placed and aligned onto the substrate, before embedding them into the core silicone with a subsequent spin‐coating step (Figure 3Aii). Laser structuring is then applied to form the desired waveguide structure from the bulk material (Figure 3Aiii). A following spin‐coating with the cladding material then fully encloses the core. After alignment, a second laser structuring defines the final outline of the waveguide (Figure 3Aiv). Adequate adhesion of the different silicone layers is achieved by oxygen plasma activation directly prior to the next spin‐coating step. The finished waveguides can then be manually peeled from the substrate (Figure 3Av).

As an alternative, we have developed a new molding approach, partly based on cleanroom microfabrication methods. The mold basis for the waveguide cores is built from SU‐8 which is processed with standard photolithography on silicon wafers (Figure 3Bi). Subsequently, aluminum (Al) as a sacrificial layer is deposited onto the SU‐8 structures by evaporation (Figure 3Bii). The silicon substrate features additional previously formed V‐grooves etched with potassium hydroxide (KOH) that enable an exact placement of ceramic ferrules with protruding glass fibers. These glass fibers are embedded into the silicone rubber during the molding process, which is performed manually by using a squeegee that spreads the core material in direction of the waveguide's end (Figure 3Biii). After curing, the silicone core is released from the mold by removing the Al layer either by electrochemical etching or by chemical dissolution. These allow the core to be peeled off the SU‐8 (Figure 3Biv). While both aforementioned approaches can generally be used to remove the Al layer, remaining aluminum residues are more often observed when etched by the electrochemical process.

To establish the surrounding thin cladding layer, a dip‐coating procedure into diluted Nusil MED‐1000 is established (Figure 3Bv).

The achievable waveguide dimensions depend on different factors: First, the diameter of the embedded glass fibers, enabling the tethering to the external laser source. Second, the applied laser parameters and the achievable resolution with the present laser–material combination. Third, the overall handleability which limits dimensions of the waveguides to a certain minimum.

Realized core dimensions were around 170 µm for laser‐based waveguides (with cladding up to around 260 µm) and range from 130 to 200 µm (cladding added around 30 µm) for mold‐based ones. For the latter, handling was compromised for larger dimensions, due to the resulting aspect ratio.

A major advantage of the laser‐based approach is its capability of rapidly changing the waveguide dimensions and overall shape. In comparison to the mold‐based approach, it is an inexpensive technique that does not need as highly sophisticated machinery and infrastructure. Drawbacks to the approach could be the sidewall roughness of the core due to laser ablation, which could negatively affect the overall optical properties. The mold‐based approach on the other hand requires a cleanroom environment and remains in a fixed design stage or needs a comparably expensive and time‐consuming turnover time for any changes. Since the sacrificial Al layer is cytotoxic, further testing of eventual residues after core release is absolutely required. Advantageous are the high precision, the accurate and easy alignment of the different layers, as well as the possibility to realize highly miniaturized waveguides with smooth core sidewalls resulting from the SU‐8 mold.

### 2.3 Characterization of Waveguides

Aiming toward a first proof‐of‐concept in vivo experiment with the fabricated waveguides, samples that were in contact with cytotoxic substances underwent a cell viability test. As an increased surface roughness at the interface of different waveguide materials affects the light propagation properties, the surface roughness of the waveguides realized with the two presented fabrication methods are compared, before the overall losses associated with each method are evaluated.

#### 2.3.1 Cell Viability

Al residues are likely to occur for both molding release procedures (electrochemically and chemically released). Besides Al residues, the etching solution used to dissolve the sacrificial layer can also affect the cell viability due to reactants and microscopic residues. As the samples fabricated with the laser‐based process are not in contact with cytotoxic materials during fabrication, these were excluded from the evaluation, and only molded samples were further examined.

Cell viability tests describe the identification of the ratio of living (able to maintain homeostasis) and dead cells in a given sample. Cell viability is the determination of living and dead cells, based on a total cell amount sample. Viable cells show the ability to maintain homeostasis, i.e., proliferation, differentiation, adaptability, and apoptosis. Cytotoxicity of the chemical aluminum etching procedure was tested by standard in vitro methods using L929 mouse fibroblasts in accordance to DIN ISO 10993‐5 (*Biological evaluation of medical devices – Tests for in vitro cytotoxicity)*, and assessed using two different methods: By live/dead staining and an alamarBlue assay.

**Figure 4:**
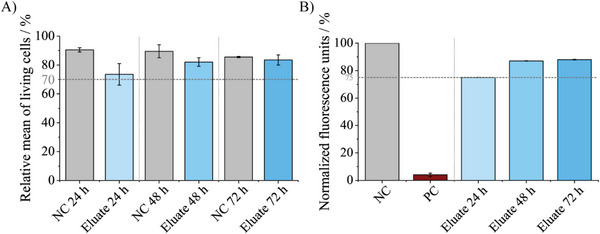
A) Normalized cell viability live/dead staining; NC = negative control, serum‐free media. B) AlamarBlue assay; NC = negative control, 100% reduction of dye, PC = positive control, cells dead (with TritonX‐100; 20%). All values were normalized to the measured fluorescence of the NC. Data are shown as mean ± standard deviation.

The live/dead staining showed 73.5% of living cells in extracts of chemically released samples after 24 h, with an increasing tendency reaching 83.5% at 72 h. All of these measurements are well above the defined limit of 70%, hence confirming no cell toxicity in accordance with ISO norms (**Figure** **4**A). In comparison, the negative control NC (serum‐free media) showed a relative mean of living cells over 85% during the entire experimental period of time.

The alamarBlue assay reached 75% of the measured normalized negative control (100%, completely reduced alamarBlue reagent) fluorescence for the eluate treated cells after 24 h (Figure 4B). An increase in fluorescence, indicative of intact metabolic activity, was observed after 48 and 72 h. The positive control PC (Triton X‐100 treated cells) was determined to be at 4% of the normalized fluorescence units of the negative control, confirming the validity of the performed test.

According to definitions of the DIN ISO 10993‐5, values of more than 70% living cells after 24 h in the live/dead staining show that the cells seem to be not critically affected by substances of waveguide manufacturing. Apart from the used chemicals for dissolving aluminum, cytotoxicity most likely results from visible or nonvisible aluminum residues on the core material of the waveguides that may be transferred into the eluate. Hence, an additional dip coat in Al80 should be implemented to eliminate further aluminum residues. This might remove any aluminum that is left behind and should leave no residues due to the ion impermeability of PDMS.

#### 2.3.2 Surface Investigation

The surface quality of the interface between core and cladding is of great importance. With high roughness present, the surface normal is significantly tilted, leading to altered impact angles of guided light that is then partially lost through the cladding. To determine the surface quality of the core silicone, both the root mean square (*R*_q_) and maximum height roughness (*R*_z_) were investigated between different manufacturing steps (**Table** **1**). For the laser‐based process, this includes the roughness determination of spin‐coated and laser‐structured surfaces (cf. Figure 3A). For the molded and released cores, the SU‐8 mold is analyzed in addition to the silicone waveguide core (cf. Figure 3B).

**Table 1:** Surface roughness values (mean ± standard deviation) of the waveguide core silicone and structures at different manufacturing steps for the two prior presented fabrication methods. Columns with final core roughness are highlighted in bold.

Roughness value	Laser‐structured core		Molded and released core			
	Spin‐ coated silicone	Laser‐structured silicone	SU‐8	Al on SU‐8	Silicone‐releasing procedure	
					Chemical (Al80)	Electrochemical
*R_q_* (nm)	65 ± 49	2800 ± 1700	2 ± 1	2 ± 0	157 ± 103	244 ± 43
*R_z_* (nm)	–	–	5 ± 2	5 ± 1	350 ± 218	499 ± 94
*n*	14	22	6	4	10	8

In summary, *R*_q_ and *R*_z_ were measured to be ranging from a couple hundreds of nanometers to a few micrometers. However, variabilities have been determined both when comparing the different manufacturing techniques as well as within one process, when roughness was measured at different process steps.

The laser‐structured core silicone showed values around 2800 nm ± 1700 nm, whereas surfaces of spin‐coated and cured core silicone only obtained *R_q_* values of 65 nm ± 49 nm. The mold itself is crucial to the quality of the molding process, SU‐8 and Al‐coated SU‐8 were investigated in addition to the released core silicone. The mold itself obtained very low roughness values, with R_q_ = 2 nm ± 1 nm for SU‐8 and 2 nm ± 0 nm for Al on SU‐8. These values did not entirely transfer onto the molded silicone core, where depending on the release procedure *R_q_* values in the range of 157 nm ± 103 nm (chemical release) and 244 nm ± 43 nm (electrochemical release) were obtained.

Hence, for both presented manufacturing approaches, the increase in surface roughness is mainly determined by the chosen processes themselves. The laser process could only be marginally influenced by optimization of parameters: on the one hand due to underlying cladding, which has a higher absorption than the core material, thus limiting the core structuring. On the other hand, laser processing of transparent silicones is a rather complex procedure itself, mainly due to the photochemically induced bond dissociation and photothermal processes.^[^ ^21^, ^24^ ^]^ Hence, melting due to the thermal effect and thus undefined topographies cannot be avoided.

The main part of the molding approach is executed manually by applying pressure onto the uncured silicone and the mold with a squeegee, which could potentially lead to a nonperfect adaption to the mold. More likely, however, is that the etching procedures affect the surface quality in the hundred nanometer range, and hence limit the smoothness of the silicone. Moreover, the curing procedure and material parameters themselves may already reach their limits. This could explain why the top surface that was in direct contact with the squeegee is not distinguishable from the sides that were in contact with the mold. Another factor to consider are remaining aluminum residues on the surface after successful release procedure. However, the top surface that was not in contact with the aluminum but only with the squeegee cannot be affected by this.

Comparing the two presented manufacturing technologies, the core‐releasing procedure gives an around tenfold lower surface roughness value than the subtractive laser‐structuring process.

#### 2.3.3 Characterization of Optical Losses

**Figure 5:**
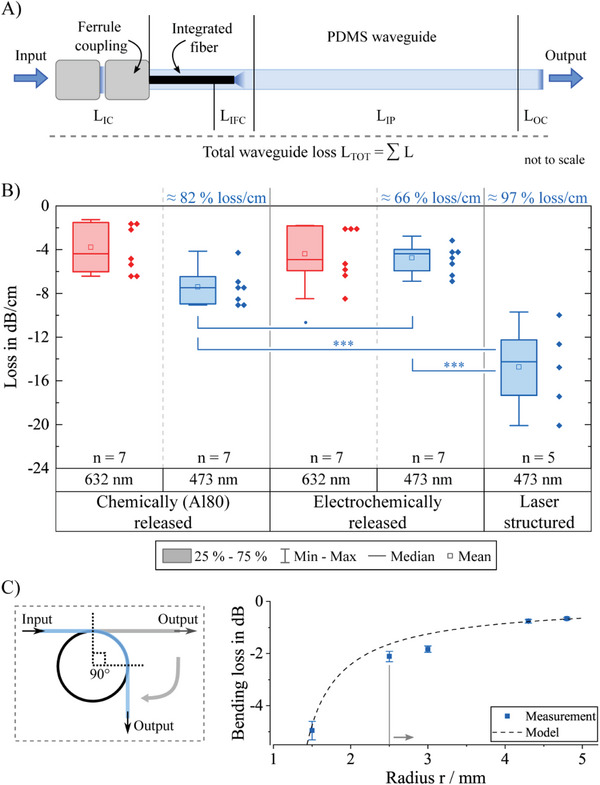
A) Total optical loss from ferrule to waveguide facet, including input coupling loss *L*_IC_, intrinsic fiber coupling loss *L*_IFC_, intrinsic path loss *L*_IP_, and the output coupling loss *L*_OC_. B) Total measured losses per length for both sacrificial aluminum approaches from coupled laser light with 632 nm (wavelength to activate red‐light sensitive opsins) and 473 nm. For both chemical (Al80) and electrochemical released waveguides seven individual samples were investigated. Losses of laser‐structured waveguides are presented only for a wavelength of 473 nm and five individual samples. Due to the limited sample numbers the individual results are plotted next to the boxes. ***p < 0.001, *p* > 0.05. C) Bending loss in relation to straight waveguide for one chemically released waveguide with 200 µm core, measured three times at 473 nm. The model (dashed line) is valid for bending radii down to the vertical line, i.e., 2.5 mm.^[^ ^27^ ^]^

The total optical loss of the waveguide is the sum of all losses from the ferrule to output facet of the silicone waveguide (**Figure** **5**A). It is divided into the input coupling loss *L*_IC_ which exists due to ferrule coupling between the multimode laser cable and integrated fiber, the intrinsic fiber coupling loss *L*_IFC_ which is defined by the fiber's numerical aperture NA and critical angle of the silicone waveguide, the intrinsic path loss of the silicone waveguide *L*_IP_ defined by the light that is dissipated during light guidance and the output coupling loss *L*_OC_ that exists due to the change in refractive index at the output facet. The intrinsic path loss of the integrated glass fiber can be neglected due to its short length and low propagation loss.

The most important loss that can be tuned during fabrication and assembly is the intrinsic path loss *L*_IP_, which is mainly determined by the surface roughness of the core material. Applying the ray optic model, an increased surface roughness leads to different impact angles of the light rays on the core/cladding interface and increases light guiding losses,^[^ ^25^ ^]^ as the critical angle of 74.2° (derived from *n*_core_ = 1.473 and *n*_cladding_ = 1.417, see Figure 2E,F) is altered.

The mean intrinsic path loss *L*_IP_ for chemically (Al80) released waveguides was measured as −3.8 dB cm^−1^ ± 2.0 dB cm^−1^ and −7.3 dB cm^−1^ ± 1.6 dB cm^−1^ for a wavelength of 632 and 473 nm, respectively, which equated to a total loss of 82% cm^−1^ at 473 nm. For electrochemical released waveguides values of −4.4 dB cm^−1^ ± 2.4 dB cm^−1^ and −4.8 dB cm^−1^ ± 1.3 dB cm^−1^ for a wavelength of 632 and 473 nm, respectively, equivalent to a total loss 66% cm^−1^ at 473 nm, were observed. The mean *L*_IP_ for laser‐structured waveguides was determined as −14.7 dB cm^−1^ ± 3.6 dB cm^−1^ for a wavelength of 473 nm, translating to a total loss of 97% cm^−1^ (Figure 5B). When analyzing all three groups at 473 nm, results show a significantly (*p* < 0.001) less loss/cm for chemically and electrochemically released waveguides, both compared to laser‐structured ones. No significant difference (*p* > 0.05) was determined between chemically and electrochemically released waveguides. The losses associated with bending of a chemically released waveguide with a 200 µm core were determined at a 90° angle for different radii and are given with respect to the straight waveguide (Figure 5C). For bending radii >4 mm, the additional loss amounted to less than −1 dB and to less than −2 dB for a bending radius of 3 mm. The theoretical bending loss (Figure 5C, dashed line)^[^ ^26^ ^]^ has been derived from a model which had already proven to match experimental data well.^[^ ^22^, ^27^ ^]^

The intrinsic path loss is mainly limited by the absorption in the waveguide material and will always occur. Based on known thickness and transmittance data of the core material, a minimum absorption loss of −0.55 and −0.85 dB cm^−1^ for a wavelength of 632 and 473 nm, respectively, can theoretically be reached. The intrinsic losses *L*_IP_ in our experiments were considerably higher than the theoretical minimum, which is a result of the surface roughness of the silicone core, as on the basis of the optical power measurements, all remaining coupling losses *L*_IC_, *L*_IFC_, and *L*_OC_ were determined to be −1.2 dB ± 1.6 dB (*n* = 23). For the molding approach, entrapped small voids that could not be visually detected might further increase absorption. Main causes for the coupling losses that lead to scattering or outcoupling, can either be voids near the embedded glass fiber, misalignment of the glass fiber itself, and the quality of the fiber facet. Unfortunately, the exact location of the embedded glass fiber facet could not always be unambiguously determined, resulting in the high standard deviation of the coupling losses. Despite the mean coupling loss being in the expected range, this imprecision led to positive values for individual samples, which is reflected in the large variability. However, as the coupling losses are relatively low compared to the total optical loss *L*_TOT_, it can be concluded that the optical loss is mainly determined by the intrinsic path loss *L*_IP_, which is mainly affected by the core's surface roughness, and was not biased by the exact position of the glass fiber facet.

### 2.4 In Vivo Cochlea Stimulation

Finally, to verify the functionality of flexible waveguides in vivo, we aimed to stimulate the genetically modified auditory nerve of a Mongolian gerbil, an established preclinical model for optogenetic hearing restoration.^[^ ^28^ ^]^ Recent approaches for optogenetic hearing restoration successfully employed optical stimulation of spiral ganglion neurons (SGNs) using conventional fibers.^[^ ^29^, ^30^ ^]^ However, compared to glass‐based fibers, the waveguides developed in this study provide not only improved structural biocompatibility, but also have novel application potential due to their bendable nature and resulting high flexibility.

**Figure 6:**
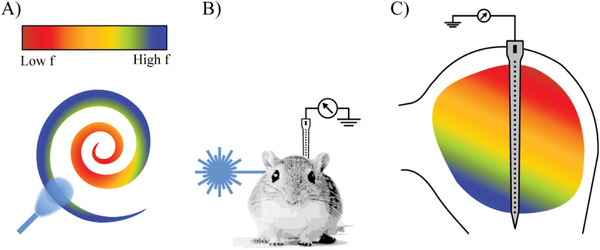
Illustration of tonotopy in cochlea and IC. A) Optogenetic stimulation was performed via the round window at the cochlear base, where high‐frequency (high *f*) tuned neurons are located. B) Optical stimulation was performed contralateral to the recording site of the silicon probe. C) Tonotopic organization of the ICC and orientation of the implanted silicon probe. Frequency *f* tuning is indicated by color, and matches the locations in the cochlea. Figure adapted from ref. [29] under terms of the CC‐BY license (BY 4.0, http://creativecommons.org/licenses/by/4.0).

As both the cochlea and the central core of the inferior colliculus (ICC) are organized in a tonotopic manner, cochlea excitation upon optogenetic stimulation (**Figure** **6**A) can be estimated by recording neural activation in the ICC using multielectrode arrays (Figure 6B,C).

**Figure 7:**
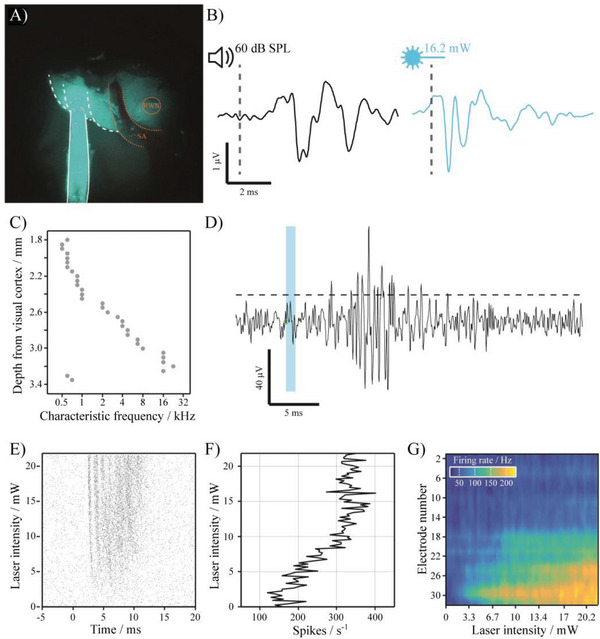
A) Surgical situs: White dashed lines indicate the coiled structure of the cochlea, while the continuous white line highlights the waveguide. Orange dotted line: stapedial artery (SA). Orange circle: round window niche (RWN). B) aABR in response to an acoustic click (0.3 ms, 60 dB SPL; left) and oABR in response to a laser pulse (1 ms, 16.2 mW) delivered via the silicone waveguide (right). Both traces present the mean of 1000 stimulus presentations at a rate of 10 Hz. C) Tonotopic order of the ICC to confirm electrode placement. The two ventral‐most electrodes are most likely located outside of the IC's tonotopically ordered central nucleus. D) Example trace of multiunit activity recorded at electrode 30 in the IC in response to a laser pulse (1 ms, 22 mW). The dotted line indicates the threshold for spike detection (median + 3 median absolute deviations). E) Scatter plot of a multiunit in response to optical stimulation of varying intensity. Individual dots indicate multiunit spiking relative to stimulus onset (0 ms). F) Firing rate of multiunits during a time window of 0–15 ms after stimulus onset (time interval set on basis of scatter plot), mean of 20 stimuli. G) Firing rate measured by different electrodes (1–32) at increasing laser intensity shown as heat map.

Upon accessing the inner ear via a retro‐auricular approach,^[^ ^28^ ^]^ an Al80‐released waveguide (−1.89 dB cm^−1^ loss, 8 mm length) was implanted into the cochlea via cochleostomy in the middle turn (**Figure** **7**A).^[^ ^29^ ^]^ To verify functional activation of the auditory system, auditory brainstem responses (ABRs, i.e., summed potentials of stimulus‐evoked activity in the ascending pathway) have been recorded upon acoustic (aABR) and optogenetic stimulation (oABR). Comparable ABR amplitudes were observed in response to 60 dB SPL (sound pressure level) acoustic clicks and 1 ms light pulses of 16.2 mW (effective power at the output facet) delivered by the waveguide (Figure 7B). ABR latency was slightly shorter in the case of optogenetic stimulation, likely due to the travel time of the sound from the speaker to the ear, and the synaptic delay between the inner hair cell and the spiral ganglion neuron (which is bypassed by direct optogenetic stimulation of SGNs). To characterize neural responses in the auditory pathway resulting from optogenetic SGN stimulation in greater detail, responses of multineuronal clusters have been recorded with a linear 32‐electrode‐array placed along the dorsoventral axis of the ICC. Confirmation of array placement was achieved by presenting pure tones of different frequencies and extracting the characteristic frequency *f* at each recording site, revealing the tonotopic axis of the ICC (Figure 7C).

Subsequently, neural activity has been recorded in response to optogenetic stimulation with a flexible waveguide (Al80‐released: −7.3 dB cm^−1^ loss, 3 mm length) placed in the round window of the cochlea, i.e., stimulating the cochlear base, where SGNs are tuned to high frequencies. Neural responses in the ICC have been defined as peaks that exceed a given threshold (mean + 3 median absolute deviations of the entire data set) at each electrode (Figure 7D). Robust ICC responses have been observed in response to a range of stimulus intensities, starting at ≈5 mW (Figure 7E), where spike rates increased as a function of laser intensity (Figure 7F). When stimulating the cochlea via the round window, neural responses were mainly detected in ventral areas of the IC (Figure 7G). This is expected based on the tonotopic organization of the auditory system, where high frequencies that are encoded at the cochlear base are represented in ventral areas of the ICC (Figure 6).

While increasing the intensity of the light source can compensate for higher optical losses in the waveguide in theory, lower losses enable the use of significantly longer waveguides due to the upper limits of laser intensity in real applications. In addition, the risk of exciting light sensitive ion channels near the stimulation site through outcoupled stray light at the waveguide's sides is lower for samples with lower intrinsic path *L*_IP_ loss.

As light intensity was the only variable parameter in our experiments, it can be assumed that the variation in spike rates is mainly driven by the radiant flux at the stimulation site. Hence, these results could be reproduced with other silicone (or glass‐based) waveguides, given the laser intensity is adjusted accordingly. These results demonstrate adequate light guidance and directed outcoupling of silicone waveguides, as optically evoked neural activity matched the expected tonotopic pattern of the auditory system.

## 3 Conclusion

The presented PDMS‐based waveguides demonstrate their ability to transmit light sufficiently in vitro also over extended periods of time and showed that they are capable of initiating responses in a first proof‐of‐concept in vivo study. Therefore, it was shown that it is possible to fabricate functional waveguides with materials well‐known from implant manufacturing, providing a potential path to translational research toward human applications, whilst achieving comparable results to other PDMS‐based devices (Comparison to other approaches in literature: Table S1, Supporting Information). Hence, flexible silicone waveguides with mean losses of −4.4 dB cm^−1^ ± 2.4 dB cm^−1^ and −4.8 dB cm^−1^ ± 1.3 dB cm^−1^ for wavelengths of 632 and 473 nm, respectively, were successfully fabricated with the electrochemical etching approach. Another entirely PDMS‐based manufacturing approach that relies on a sophisticated cleanroom process obtained a loss of −4.7 dB cm^−1^ at 1550 nm with a 50 µm waveguide core,^[^ ^18^ ^]^ whereas a method that uses PDMS as core material and the surrounding medium as cladding had a fraction of 31% ± 10% of input light at the fiber tip.^[^ ^31^ ^]^ PDMS‐based waveguides have also been successfully integrated into lab‐on‐chip devices achieving reported losses of −3.1 dB cm^−1^ at 532 nm and −2.9 dB cm^−1^ at 633 nm,^[^ ^32^ ^]^ whereas a different approach even reached down to −0.40 dB cm^−1^ at 460 nm.^[^ ^33^ ^]^ Both approaches feature substrate bound samples that are hard to isolate and integrate in other assemblies, compromising different applications of these waveguides. Combined Parylene C and PDMS waveguides where the Parylene C core was etched to dimensions around 30 µm obtained −6.1 dB cm^−1^ at 450 nm.^[^ ^34^ ^]^ Many other approaches use materials such as hydrogel or polycarbonate. A combined PDMS/hydrogel fiber fabricated by thermal drawing achieved losses of only −1.018 dB cm^−1^,^[^ ^20^ ^]^ being comparable to another hydrogel‐based fiber with losses of −1.07 dB cm^−1^.^[^ ^35^ ^]^ Thermal drawing of PC/COC fibers achieved core dimensions around 100 µm and had losses of −1.9 dB cm^−1^ at 473 nm.^[^ ^36^ ^]^

Whilst the obtained losses of the presented study cannot compete with hydrogel‐based devices, major advantages of the presented waveguides are first, the availability of PDMS as medical grade materials, which is so far not given, e.g., for hydrogel, emphasizing the path to translational research with PDMS‐based waveguides. Second, the low Young's modulus of PDMS, especially when compared to conventional glass fibers or glass fiber materials, which are additionally prone to breakage and to potentially cloud or dissolve over time.^[^ ^37^, ^38^ ^]^

Future research will include further core surface investigations and potential process variations with the ultimate goal to reduce roughness, hence leading to a decrease of optical losses and therefore eventually pushing the waveguide performance into the direction of hydrogel‐based devices.

However, capitalizing on a preclinical model of optogenetic hearing restoration, we were thus already able to demonstrate that the concept of flexible, silicone‐based waveguides indeed qualifies as an alternative to rigid waveguides in biomedical applications.

## 4 Experimental Section

### Sample Fabrication for Transmission and Refractive Index Measurements

The one component cladding silicone Nusil MED‐1000 (NuSil Technology LLC, USA) was mixed with *n*‐heptane (≥99%, Carl Roth, Germany) in a ratio 1:1. It was spin‐coated (three layers on top of each other, each with 1500 rpm) on a ceramic substrate covered with tape to allow simple release of the cured silicone with ethanol. Afterward, it was left to cure at room temperature and atmospheric moisture for 10 d. The resultant thickness was measured to 460 µm ± 90 µm (*n* = 10). Two‐component (ratio 1:1) core silicone Nusil MED‐6755 (NuSil Technology LLC, USA) was cast with a PMMA mold and cured overnight in a pressurized chamber at 90 °C and 1.2 bar. Resulting layer thicknesses were measured to 1190 µm ± 120 µm (*n* = 10). For each silicone type, two batches were processed. The cured silicone foils were laser‐cut to circle‐shaped samples with a diameter of 8 mm.

### Transmission and Refractive Index Measurement

Transmittance spectra were recorded with a UV–vis spectrophotometer (Cary 500, Varian Australia Pty Ltd., Mulgrave, Vic, Australia) from 400 to 1200 nm in a 2 nm data interval. To perform the measurement the samples were placed on a clean microscope slide. To avoid substrate interference, a blank microscope slide was placed in the reference path. Both light paths were confined by a 5 mm aperture. Baseline correction with blank microscope slides was performed prior to the first and at least after every ten subsequent measurements. The refractive indices of the samples were measured with an automatic critical‐angle spectral refractometer (DSR_λ, Schmidt+Haensch GmbH & Co., Berlin, Germany) for nine different wavelengths, i.e., 404.7, 435.8, 486.1, 546.1, 587.6, 589.3, 632.8, 656.3, and 706.5 nm at 20 °C.

### Accelerated Ageing

Transmittance and refractive index samples were stored in phosphate buffered saline solution (PBS) (Merck, Germany) in an incubator (Heraeus Holding GmbH, Hanau, Germany) at 37 and 60 °C for an extended period of time. The ageing was based on first‐order chemical reactions, being described by the Arrhenius equation. For many polymers, a duplication of ageing rate for every 10 °C temperature increment was assumed.^[^ ^23^ ^]^ The accelerated ageing factor α can therefore be given as: $\alpha \;=2^{\frac{T_{2}-T_{1}}{10}}$ with *T*_2_ = 60 °C and *T*_1_ = 37 °C, resulting in α ≈ 4.9 for storage at 60 °C.^[^ ^23^ ^]^ From the results obtained at a higher temperature, a potential time of use at the lower temperature can then be extrapolated.

### Laser‐Structured Waveguides

Cladding silicone was mixed with *n*‐heptane (≥99%, Carl Roth, Germany) in a ratio 1:1, to achieve the required viscosity for fabricating thin layers. It was spin‐coated on a 2‐in. ceramic substrate covered with tape and left to cure for (at least) 3 d at ambient temperature and humidity to an about 50 µm thick film. The ceramic substrate had been covered with tape before the spin‐coating step to allow simple release of the cured silicone with ethanol. The ceramic carrier was placed in a custom chuck made of polytetrafluorethylene (PTFE) which supported the following spin‐coating steps and glass fiber fixation. A Kapton‐buffered glass fiber (FVP SI100/110/125, NA 0.22, silica/silica, high OH, polyimide buffer, MM, 180–1150 nm, Laser Components GmbH, Olching, Germany) was coated with an adhesion layer realized by plasma enhanced chemical vapor deposition (PECVD), composed of amorphous silicon carbide (SiC) and silicon oxide (SiO_x_). Together with the PDMS‐film, glass fiber pieces were activated with oxygen plasma in a plasma generator (Nano UHP‐RF‐PC, Diener electronic GmbH + Co. KG, Ebhausen, Germany). Subsequently, the glass fibers were aligned on the PDMS film and bonded in a way that they overlapped it for about 1.5 cm. The overlapping parts were covered with Kapton tape (polyimide film tape 5413, 3 M Company, St. Paul, MN) and fixed to the Teflon carrier to protect them in the posterior spin‐coating steps. Another oxygen flash followed before the core silicone was spin‐coated to a layer thickness of about 120 µm, embedding the core and cladding of the uncovered glass fiber pieces. It was left to cure overnight in a pressurized chamber at 90 °C and 1.2 bar. Afterward, the waveguide core was laser‐patterned with a Nd:YVO_4_ picosecond laser (Rapid 10, Coherent Kaiserslautern GmbH (former Lumera Laser GmbH), Kaiserslautern, Germany). Careful cleaning of ablative debris was performed with a mixture of 0.5% glass detergent (Teepol‐L, Teepol Products, Kent, UK), 2.5% sodium phosphate (≥98%, Carl Roth, Germany), and 97% ultrapure water. Compared to ethanol (or other alcohols commonly used for cleaning) this mixture, the so called “Leslie's soup,” kept swelling of the silicone rubbers low and the surfactant allowed damage‐free cleaning with a microbrush. Once the core structures were cleaned and dried an oxygen flash was applied and cladding silicone (again mixed with *n*‐heptane in a ration 1:1) was spin‐coated to a layer thickness of about 20 µm. The protective Kapton tape was released from the glass fibers and the silicone rubber was left to cure for (at least) 3 d. Subsequently the lateral outlines of the waveguides were defined by laser‐patterning with the picosecond laser. The waveguide's outlets were cut with a scalpel blade, leaving the core free from cladding at the frontal facet. These processed waveguides were carefully released of the ceramic carrier with tweezers.

### Mold Fabrication

Due to the dimensions of the glass fibers, resist thicknesses of over 125 µm must be achieved, enabling the embedding of the glass fibers into the silicone by molding. SU‐8 3050 (Kayaku Advanced Materials, Westborough, MA, USA) can be spin‐coated to form such a thick layer. SU‐8 is a highly crosslinked negative photoresist which is not meant to be removed. To ensure a placement of ferrules (CFMLC21L10, Thorlabs, Newton, NJ) onto the substrate in such a way that the optical fiber is close above the substrate's surface, deep‐etched V‐grooves in the range of half the diameter of the ferrule, namely, 625 µm, were required. Therefore, 1000‐µm‐thick wafers with {100} crystal orientation were used as substrate. Thermal wet oxidation at 950 °C was performed on bare silicon which resulted in a 300‐nm‐thick silicon dioxide (SiO_2_) layer. A low‐pressure chemical vapor deposition (LPCVD) of 100 nm silicon nitride (Si_3_N_4_) at 770 °C followed. It resulted in a 400‐nm‐thick SiO_2_/Si_3_N_4_ layer on both sides of the wafer. Photolithography using 2‐µm‐thick AZ1518 (Microchemicals GmbH, Ulm, Germany) was performed, masking the front SiO_2_/Si_3_N_4_ layer for the subsequent reactive ion etching (RIE). Rectangular structures were opened in the SiO_2_/Si_3_N_4_ that allowed to etch 700 µm deep V‐grooves by potassium hydroxide (KOH 45%, Oqema, Germany, thinned down to 30%) wet etching. For that purpose, the wafers were immersed in 30 wt% KOH solution at 80 °C and left there for 8 h 40 min. Afterward, the SiO_2_ and Si_3_N_4_ layers were removed with hydrofluoric acid (HF 50%, Technic France, France).

The molding approach used two layers of SU‐8 3050 to obtain an overall structural thickness of 135 µm. The first layer was spun on with 1450 rpm for 30 s and baked at 95 °C for 45 min resulting in a thickness of ≈95 µm. The second layer was spun onto the substrate with the same parameters but at 2900 rpm and a subsequent bake at 95 °C for 30 min resulting in a thickness of ≈40 µm. The exposure was performed for 70 s at 9 mW cm^−2^ utilizing an i‐line filter to block UV radiation below 350 nm.^[^ ^39^ ^]^ Then, a postexposure bake was performed for 5 min at 95 °C. The development was completed using the chemical mr‐Dev 600 (micro resist technology GmbH, Berlin, Germany). A hard bake was performed at 125 °C for 25 min increasing the crosslinking of the resist and helping to level the resist surface. Every baking step was done on a leveled hotplate with a temperature ramp of ≈3 °C min^−1^ to reduce thickness variation over the wafer.

An aluminum (Al) layer was evaporated to a thickness of 300 nm (Leybold Univex 500, Cologne, Germany). This layer acts as a sacrificial layer to remove the PDMS waveguides in both chemical and electrochemical release procedures. At the end of the process, molds were available in which the silicone can be molded directly. However, for the electrochemical etching approach another conducting layer of 200 nm tungsten titanium (WTi) was deposited beneath the aluminum by radio frequency (RF) sputtering at 100 W (Leybold Univex) in seven cycles with 5 min process and 1 min wait time prior to the aluminum evaporation step.

### Waveguide Core Molding

To ensure a strong adhesion between glass fiber and silicone‐rubber, the glass fiber surface was activated with an O_2_‐plasma at 80 W and 13.56 MHz in a plasma reactor (Nano UHP‐RF‐PC, Diener electronic GmbH + Co. KG, Ebhausen, Germany). For that purpose, the ferrules were taped on a carrying substrate, leaving the glass fiber that was cleansed with ethanol exposed on all sides. After O_2_‐plasma activation, the ferrules were placed inside the KOH etched V‐grooves where they self‐aligned themselves, centering the glass fibers in the photolithographic‐structured rectangular resist trenches. By using a pneumatic dispenser, the core silicone was dispensed near the ferrules. Then the silicone rubber was spread in direction of the waveguides’ ends with the help of a squeegee (serilor SR1 P0 75 shA, H.‐D. Buschkamp, Gütersloh, Germany) by applying mechanical pressure. The curing of the silicone was done in a pressurized chamber at 70 °C and 1.2 bar for 3 h.

### Electrochemical Aluminum Dissolution

Process parameters were selected based on previously reported research.^[^ ^40^, ^41^ ^]^ The wafer that carried the mold with the silicone core material and the stack of WTi and sacrificial aluminum was electrically contacted with crocodile clips. An alumina ceramic coated with sputtered platinum was used as counter electrode. Both the wafer and the ceramic were immersed in a 0.01 mol L^−1^ NaCl solution (>99%, Merck Supelco, Germany) which was used as electrolyte for the reaction. The platinum electrode was connected to the negative while the wafer is connected to the positive output of the triple power supply HM7042‐5 (HAMEG Instruments (Rohde & Schwartz), Munich, Germany). A voltage of 1.6 V was applied to start the electrochemical dissolution of the aluminum layer surrounding the core silicone and the resulting current was observed. After 6 h, when no visible aluminum was left on the wafer and no bubbles were developed on the substrate, the wafer was disconnected. While the mold was immersed in DI water, the waveguides were manually peeled from the SU‐8 trenches using tweezers.

### Chemical Aluminum Etching

The chemical TechniEtch Al80 (referred to as Al80 in the following, MOS quality purity grade, MicroChemicals GmbH, Ulm, Germany), a commercially available etching solution consisting of phosphoric acid (H_3_PO_4_), nitric acid (HNO_3_), acetic acid (CH_3_COOH), and water (H_2_O) in a ratio of H_3_PO_4_:HNO_3_:CH_3_COOH:H_2_O = 73.0%:3.1%:3.3%:20.6% was heat up to 77 °C.^[^ ^42^ ^]^ The wafer was immersed in the acid for 6 h. After no visible aluminum residues were left on the surface the wafer was rinsed with DI water and left in a petri dish in clean DI water over night to make sure acid residues are removed. While the wafer was immersed in DI‐water, the flexible waveguides were peeled from the SU‐8 molds using tweezers.

### Waveguide Cladding Dip Coat

The waveguide cores were cleansed with ethanol and then plasma activated with an O_2_‐plasma with the same parameters as in the silicone molding process. *n*‐heptane and cladding silicone were mixed in a ratio of 3:1 to obtain a low viscous silicone bath. The ferrules were taped to a ceramic, exposing the silicone ends over the ceramic edge. Afterward, the waveguides were dipped inside the liquid silicone and were pulled out immediately with a horizontal movement which forms a thin cladding film. The dip‐coated waveguides were left hanging for 15 min until cured adequately for storage. To expose the waveguide core, excess silicone was cut from the tip with a razorblade resulting in a finished flexible waveguide.

### Optical Characterization

To estimate the intrinsic path loss *L*_IP_ of the flexible waveguides, the cut back method was used. The waveguides were connected to lasers with 473 nm (10 mW, eLas educational lasers (former miCos, Buggingen, Germany)) and 632 nm (15 mW HeNe, Melles Griot GmbH, Bensheim, Germany) wavelength, respectively, in vertical orientation to avoid mechanical load on the flexible silicone. The initial output power and length of the individual waveguides were determined, and afterward 2 mm pieces were manually cut repeatedly from the tip of the flexible waveguides with a razor blade. A sliding caliper was used for the length measurements. The manual cut was performed with an accuracy of 2.0 mm ± 0.2 mm which was determined by investigation of cut silicone pieces with a light microscope and measuring software. It was taken special care to perform the manual razor blade cuts in an unvaried way for the period of the measurements. The optical output power was measured after each cutting step. A rise in power can be measured from which the light loss inside the silicone is calculated by the resulting slope of the measurement curve. The resulting loss *L* in dB is then calculated by $L\;=\;10\cdot \mathrm{lo}g_{10}\left(\frac{P_{\mathrm{out}}}{P_{\mathrm{in}}}\right)$ with *P*_out_ the measured power at the tip of the waveguide and *P*_in_ the input power without connected waveguide. A FC/PC to ferrule patch cable (M61L01, Thorlabs, Newton, NJ) was used as adapter to connect a 2.5 mm diameter ferrule laser cable (M122L01, Thorlabs, Newton, NJ) to an optical fiber with NA of 0.22 and 200 µm core diameter to the 1.25 mm silicone waveguide ferrule with an integrated fiber with 105 µm core diameter and NA of 0.22 as well. The OMM‐6810B optical multimeter and OMH‐6722B silicon power/wavehead (ILX Lightwave Corporation, Bozeman, MT) were used to measure the optical power. The wavelength was set to the previously described values of the used red and blue lasers. The optical ferrule of the waveguide was connected to the multimode patch cable by using a postmountable ferrule clamp and the ADAL3 interconnect (Thorlabs, Newton, NJ).

To measure the bending loss at a 90° angle, the waveguides were placed on sealing rings with different diameter thus providing defined radii. The waveguides were aligned on the surface and then connected to the already described optical power measurement setup.

### Statistics

A standard analysis of variance (ANOVA) was performed to statistically compare the optical loss results from the different manufacturing approaches to each other. A post hoc Tukey's test was used to compare all possible combinations. Due to low sample size, the *η* ^2^ global effect size was determined, too, resulting in a high value of 0.76.

### Surface Investigation

An optical white light profilometer (Wyko NT9100, Veeco, Plainview, NY) was used to investigate the surface roughness of both the photoresist molds, the released core silicone material and the laser‐structured silicone cores. The photoresist surface was measured before and after hard baking. In addition, the surface roughness after metal deposition was measured and compared to the roughness values of the silicone after release from the trenches of the mold. Surfaces from the laser structuring process were measured after spin‐coating as well as after laser patterning. The mode was set to phase‐shifting interferometry (PSI) with a set amplification of 50 leading to an effective magnification of 50.569 to investigate a surface of (0.125 × 0.094) mm^2^. The silicone surfaces were measured in the vertical scanning interferometry (VSI) mode with the same magnification. Root mean square *R*_q_ as well as the maximum height *R*_z_ (from valley to peak) were determined.

### Extract Preparation for Cell Viability Assay

Tests were conducted in accordance with DIN ISO 10993‐5:2009. Here, 0.450 g silicone core material in 2 mL serum‐free Dulbecco's modified Eagle medium (DMEM, Thermo Scientific, Schwerte, Germany), 2 × 10^−3^ m l‐alanyl‐l‐glutamine (Thermo Scientific, Schwerte), and 0.1 mg mL^−1^ kanamycin (Sigma‐Aldrich, Steinheim, Germany) were used. Incubation of extracts was carried out for 48 h at 37 °C and 5% CO_2_. Samples were then set aside protected from light at 8 °C for maximum one week.

### AlamarBlue Assay

Cytotoxicity and cell viability were quantitatively assessed using the alamarBlue assay (Bio‐Rad Laboratories GmbH, Munich, Germany). A plate reader with 560 nm excitation and 590 nm emission wavelength was used to measure the relative fluorescent units (RFU) of the medium supernatant for samples, and positive and negative controls. The test was performed twice at three different time points (24, 48, and 72 h) on three independent biological samples. Therefore, 10% alamarBlue solution was added to the extracts. Subsequently, incubation of the cells took place for four hours at 37 °C in a 5% CO_2_ atmosphere in the dark prior to the collection of the supernatant. In addition to the samples, negative and positive controls were prepared. The negative control, i.e., 100% reduction of resazurin, consisted of a 10% alamarBlue solution in growth medium that was autoclaved. Cells were incubated in 20% Triton X‐100 solution (Sigma‐Aldrich, Germany) for 10 min and stained to establish the positive control.

### Live/Dead Staining

To rapidly quantify the amount of viable cells, life/dead staining and fluorescence read‐out on three independent samples was performed. For labeling and later identification of the viable cells, the green fluorescent nucleic dye SYTO 16 (Life Technologies, Carlsbad, CA, 5 × 10^−6^ m) was used. For identifying the dead cells, propidium iodide (PI) (Sigma‐Aldrich, Steinheim, Germany, 1.5 × 10^−9^ m) in DMEM was used. The live/dead staining was performed by 20 min incubation of the dyes (concentration), after washing the cells with PBS at the three different timepoints (24, 48, and 72 h). Cells (100 000 fibroblasts in 200 µL growth medium) incubated with the eluates were then seeded and incubated for 24 h. The medium was removed after 24 h of incubation and replaced by 200 µL of each extract which were each changed after 24 h. Two samples of cells cultured in serum‐free medium were used as negative control for each time point.

### In Vivo Optogenetic Stimulation of the Auditory Nerve

The animal experiment was performed in compliance with the German national animal care guidelines and approved by the animal welfare offices of the University Medical Center Göttingen and of the state of Lower Saxony, Germany (LAVES, License No. 17‐2394). The experiment was performed in a sound‐proofed chamber under isoflurane anesthesia (4% at 1 L min^−1^ for induction, 1%−2% at 0.4 L min^−1^ for maintenance). Applying buprenorphine (0.1 mg kg^−1^ body weight) subcutaneously realized adequate analgesia. A heating pad maintained body temperature.

The left cochlea of a Mongolian gerbil (*Meriones unguiculatus*) was injected with a viral suspension (AAV‐PHP.B) carrying DNA coding for the blue‐light‐sensitive ChR2‐variant *CatCh* under control of the human synapsin promotor (titer: 6.99 × 10^12^ genome copies mL^−1^) to render the auditory nerve light sensitive as described before.^[^ ^28^ ^]^ After a period of several weeks (to allow for recovery from the surgery and opsin expression in the auditory nerve), the cochlea was surgically exposed by a retro‐auricular approach.^[^ ^28^ ^]^ As described previously,^[^ ^28^ ^]^ laser‐coupled (473 nm, 100 mW DPSS; Changchun New Industry Optoelectronics, Changchun, China) waveguides were then inserted into the cochlea either via the round window or in the middle cochlear turn via a cochleostomy. Acoustic stimuli were presented from a free‐field loudspeaker. ABRs were recorded via subcutaneous needle electrodes placed in two locations (vertex and mastoid) and connected to a custom amplifier, while a subdermal active shielding electrode was inserted near the tail. For a/oABR recordings, stimuli of increasing intensity were presented 1000 times each before averaging filtered data (0.3–3 kHz Butterworth filter). Following the ABR measurements, a linear 32‐electrode silicon probe (177 µm^2^ electrode surface, 50 µm spacing between electrodes, 1−3 MΩ at 1 kHz, Neuronexus, Ann Arbor, MI) was implanted into the ICC via a craniotomy in the skull 2 mm lateral and 0.5 mm caudal to lambda to characterize neural responses in greater detail. In gerbils, the ICC was covered by the visual cortex. Therefore, the bottom electrode was navigated to its surface and then lowered to a depth of ≈3.3 mm. To obtain a stable preparation, the implant was allowed to rest for one hour. Before optical stimulation, pure tones of different frequencies and intensities were presented in order to confirm electrode placement along the tonotopic axis of the ICC. Flexible waveguides were then inserted into the cochlea through the round window, and laser pulses lasting 1 ms with different intensities were presented at a rate of 1 Hz (duty cycle of 0.1%). Data were digitized at a rate of 32 kHz (Digital Lynx 4s, Cheetah, Neuralynx Inc.). A threshold of three median absolute deviations above the mean was applied for each channel (filtered between 0.6 and 6.0 kHz) of the total trace, and the timestamps of peaks exceeding this threshold were extracted and taken for further analysis.

## Conflict of Interest

The authors declare no conflict of interest.

## Supporting information

Supporting Information

## Data Availability

The data that support the findings of this study are available from the corresponding author upon reasonable request.
