# Supplementary material for: Fabrication and Characterization of PDMS Waveguides for Flexible Optrodes
Source: Adv Healthc Mater. 2024 Apr 28;13(16):2304513. doi: 10.1002/adhm.202304513 (PMC11469164; doi:10.1002/adhm.202304513)
Supplement: Supplementary file 1 — Supporting Information [file ADHM-13-2304513-s001.docx]

| Reference no. | Materials | Fabrication | Core dimensions (width/diameter) / µm | Wavelength / nm | Loss / dB∙cm^-1^ |
| --- | --- | --- | --- | --- | --- |
| 18 | Wacker Elastosil  Core: RT601  Cladding: RT604 | Thin-film | 10 | 1550 | -4.6 |
|  |  | Molding | 50 |  |  |
| 20 | Core: PDMS  Cladding: PVA/PAA Hydrogel | Thermal drawing | 100 – 500 | blue | -1.018 |
| 31 | Core: Sylgard184  Cladding: Brain/CSF | Drawing/Pulling | 20 – 200 | 465 | -10.6 |
| 32* | Core: PDMS  Cladding: Air | Squeezed and pressed | 50 | 532 | -3.1 |
| 33* | Core: PDMS  Cladding: PDMS | SU8 mold | 125 | 460 | -0.4 |
| 34 | Core: Parylene C  Cladding: PDMS (Sylgard184) | Waveguide core etched | 30 | 450 | -6.1 |
| 35 | Core: nanocrystalline PVA hydrogel  Cladding: amorphous PVA hydrogel | Silicone tubing mold | 600 | 473 | -1.07 |
| 36 | Core: PC (Polycarbonate)  Cladding: COC  (Cyclic Olefin Copolymer) | Thermal drawing | 100 – 130 | 473 | -1.9 |
| This work | Core: MED6755  Claddng: MED1000 | Molding | 130 – 200 | 473 | -4.8 to  -7.4 |
|  |  | Laser structuring | ≈ 170 |  | -14.7 |

**Table** S1: Comparison of key parameters of polymer-based light guides for optogenetic applications.

* Waveguide integrated in Lab-on-Chip device
